# Harnessing Facebook for Smoking Reduction and Cessation Interventions: Facebook User Engagement and Social Support Predict Smoking Reduction

**DOI:** 10.2196/jmir.6681

**Published:** 2017-05-23

**Authors:** Sunny Jung Kim, Lisa A Marsch, Mary F Brunette, Jesse Dallery

**Affiliations:** ^1^ Center for Technology and Behavioral Health Department of Biomedical Data Science, Department of Psychiatry Geisel School of Medicine at Dartmouth, Dartmouth College Lebanon, NH United States; ^2^ Center for Technology and Behavioral Health Department of Psychiatry Geisel School of Medicine at Dartmouth, Dartmouth-Hitchcock Lebanon, NH United States; ^3^ Department of Psychology University of Florida Gainesville, FL United States

**Keywords:** social media, social support, behavior and behavior mechanisms, smoking cessation, persuasive communication, social networking, technology, health promotion

## Abstract

**Background:**

Social media technologies offer a novel opportunity for scalable health interventions that can facilitate user engagement and social support, which in turn may reinforce positive processes for behavior change.

**Objective:**

By using principles from health communication and social support literature, we implemented a Facebook group–based intervention that targeted smoking reduction and cessation. This study hypothesized that participants’ engagement with and perceived social support from our Facebook group intervention would predict smoking reduction.

**Methods:**

We recruited 16 regular smokers who live in the United States and who were motivated in quitting smoking at screening. We promoted message exposure as well as engagement and social support systems throughout the intervention. For message exposure, we posted prevalidated, antismoking messages (such as national antismoking campaigns) on our smoking reduction and cessation Facebook group. For engagement and social support systems, we delivered a high degree of engagement and social support systems during the second and third week of the intervention and a low degree of engagement and social support systems during the first and fourth week. A total of six surveys were conducted via Amazon Mechanical Turk (MTurk) at baseline on a weekly basis and at a 2-week follow-up.

**Results:**

Of the total 16 participants, most were female (n=13, 81%), white (n=15, 94%), and between 25 and 50 years of age (mean 34.75, SD 8.15). There was no study attrition throughout the 6-time-point baseline, weekly, and follow-up surveys. We generated Facebook engagement and social support composite scores (mean 19.19, SD 24.35) by combining the number of likes each participant received and the number of comments or wall posts each participant posted on our smoking reduction and cessation Facebook group during the intervention period. The primary outcome was smoking reduction in the past 7 days measured at baseline and at the two-week follow-up. Compared with the baseline, participants reported smoking an average of 60.56 fewer cigarettes per week (SD 38.83) at the follow-up, and 4 participants out of 16 (25%) reported 7-day point prevalence smoking abstinence at the follow-up. Adjusted linear regression models revealed that a one-unit increase in the Facebook engagement and social support composite scores predicted a 0.56-unit decrease in cigarettes smoked per week (standard error *=*.24, *P*=.04, 95% CI 0.024-1.09) when baseline readiness to quit, gender, and baseline smoking status were controlled (*F*_4, 11_=8.85, *P*=.002).

**Conclusions:**

This study is the first Facebook group–based intervention that systemically implemented health communication strategies and engagement and social support systems to promote smoking reduction and cessation. Our findings imply that receiving one like or posting on the Facebook-based intervention platform predicted smoking approximately one less cigarette in the past 7 days, and that interventions should facilitate user interactions to foster user engagement and social support.

## Introduction

### Background

Tobacco use is the primary cause of premature mortality and is responsible for almost half a million deaths every year in the United States and nearly 5 million deaths globally [1]. Although mass media–based (eg, TV, radio) health campaigns have been moderately effective for health promotion—influencing 4% to 8% of the population to change health behaviors [2]—several considerable drawbacks remain. First, mass media–based approaches for public health promotion, such as tobacco control and prevention campaigns, fall short in enabling a frequent and durable delivery system that can disseminate individually tailored messages. Second, a message exposure tracking system is often nonexistent or costly in traditional mass media settings. Thus, it is difficult to directly gauge the effects of mass media exposure to health messages on health behavior. Third, traditional media platforms offer a limited to nonexistent interactive engagement system that could enhance social support and user-centered engagement.

Significant advances in social media technologies and their ubiquity offer novel opportunities to provide geographically distant users with easily accessible, cost-effective, personalized health content, and social network-based support. For example, Facebook, one of the most widely adopted social media platforms, hosts approximately 1.22 billion daily active users [3] and has an enormous quantity of user-initiated virtual communities that are highly relevant to people seeking social support for health problems [4]. The magnitude of this social network platform and its popularity can considerably extend the reach of evidence-based health messages to the public and scale-up user-centered social support to the population level to address public health problems. In fact, a growing volume of research is leveraging social media to facilitate health behavior changes such as increasing physical activities [5], enabling addiction recovery support [6], providing support for cancer survivors [7,8], and reducing sexual risk behaviors among youth [9]. More evidence has shown that participants perceive social media approaches for health promotion as appealing, acceptable, and convenient [10].

In this regard, social media such as Facebook provide a range of communication features for putative processes of behavior change that are important to individuals with health problems. Those social media features and related processes include “posting” features for self-disclosure [11], search functions via hashtags for information-seeking [12], “share” features for social sharing [13], and using “comment” and “reaction” features for engagement and social support [14-16]. Strategic use of social media features that facilitates these processes may foster desirable health outcomes [17].

Facebook groups, in particular, can be used as a designated online social support community for members with similar health concerns [18]. They provide various social interaction features such as “likes” and “comments” for group members. Feedback “comments” from group members and clinicians as well as reciprocated interactivities can create a supportive environment for achieving health promotion [4]. Researchers can also track whether the target audiences view the intervention content and observe social dynamics among group members in adopting health attitudes and behaviors.

In this study, we utilized Facebook group features to effectively disseminate prevalidated antismoking messages with high frequency and longitudinal exposure. Message exposure frequency and exposure duration are pivotal factors for successful health campaigns [19] that are often limited in traditional mass media environments. In addition, we harnessed multidirectional communication processes among intervention target participants (smokers) to foster user-centered engagement and social support.

Despite the potential benefits of harnessing social media for health interventions, a critical gap in knowledge persists in terms of how to best utilize social media features to achieve positive health intervention outcomes. The intent of this study is to strategically leverage communication features that are available on Facebook groups to implement a smoking reduction and cessation intervention among regular smokers who are interested in quitting. For our intervention, we promoted smoking cessation as the optimal outcome to achieve, but we also accepted smoking reduction as a positive change for those who could not immediately quit smoking, as smoking reduction is a common step toward eventual cessation [20]. In doing so, we designed a smoking reduction and cessation intervention through a Facebook group with two primarily theory-guided intervention components: (1) message exposure to antismoking media content in a systematic manner while strategically changing messaging frequency (high vs low), and (2) social support and engagement systems with different levels (high vs low).

### Exposure to Antismoking Messages

Prior studies on health promotions and health behavior models have demonstrated that exposure to health communications can enhance one’s health behavior by changing core beliefs and attitudes about expected health outcomes [21], by providing education on the skills needed to change health behavior [22], and by disseminating knowledge about the target health behavior [23]. In realizing these intermediate factors for behavior change, acquiring exposure to intervention messages is the most important requisite condition for successful health promotion [24]. This study targeted systematic, frequent exposure to antismoking messages as a key factor. We posited that strategic exposure to antismoking messages could influence the target audience to form positive beliefs and behaviors about smoking reduction and cessation [25]. Different from traditional media environments, leveraging social media as a communication platform for a health intervention enables investigators to predesign and administer the frequency and delivery schedule of message exposure in a systematic manner (eg, one-time message exposure at the same time of the day). Social media also enable interventionists to disseminate campaign messages to geographically distant audiences. For our Facebook group–based intervention messages, we aimed to use preexisting, well-received antismoking campaign messages (eg, the Tips From Former Smokers campaign) rather than generating a new set of antismoking messages. In order to reduce the cost, time, and risks associated with developing new campaign content, the CDC’s (Centers for Disease Control and Prevention) best practice guidelines for tobacco control and prevention campaigns recommend reuse of existing campaign messages that have shown positive campaign outcomes [1].

### Social Support and User Engagement

Social support and engagement systems were additional theoretical components that were applied in our Facebook group–based smoking reduction and cessation intervention. Social support is defined as informational, emotional, reassuring, or tangible resources [26] provided by professional or nonprofessional social capital. Social support has been shown to be one of the most considerable coping resources for achieving desirable health outcomes [27], including behavioral health [28], physical health [5], and mental health [29]. Social media can be an excellent outlet for active user engagement and peer-to-peer health support [30,31]. Social media technologies offer various communication and social networking features that individuals can use to share their health concerns and engage their social networks for support [30]. In the intervention, we implemented health communication strategies to deliver social support and user engagement systems, which in turn may foster smoking reduction and cessation [32]

Based on the four dimensions of the social support conceptual framework [33], we operationalized three types of social support constructs—emotional support, informational support, and reassuring support—through comments and wall postings on our smoking reduction and cessation Facebook group. For emotional support, we showed an empathic understanding of the issues of participants (eg, We know how hard it is to quit smoking, and understand what you are going through). To operationalize informational support, we appraised the participants’ circumstances and posted advising comments on topics ranging from nicotine craving symptoms to possible solutions (eg, Here are some tips for managing cravings), and we also provided reassuring support by assuring self-confidence to the participants through affirmative comments (eg, You can do it!).

One’s perceived social support can help them enhance their self-efficacy beliefs in order to overcome barriers to adopting the health behavior being promoted. To deliver social support and engagement in relation to promoting smoking reduction and cessation during the 4-week intervention period, we manipulated the level of engagement and social support systems (high vs low) and juxtaposed it with high versus low message exposure.

### Specific Objectives

We examined the feasibility of a Facebook group–based smoking reduction and cessation intervention. Additionally, the preliminary efficacy on smoking reduction (the reduced number of cigarettes consumed per week) and on 7-day point smoking abstinence at the follow-up was tested. We also tested whether the intervention components (social support and engagement systems) predict smoking reduction.

## Methods

### Recruitment

Recruitment messages and preliminary screening questions were disseminated through Amazon Mechanical Turk (MTurk) and social media platforms. MTurk is an anonymous Web-based labor market with over 500,000 registered workers worldwide. MTurk workers complete tasks distributed by requesters for small financial rewards. MTurk has been used as a recruitment pool in various fields of research for an array of tasks, including decision-making [34-36], health literacy [37], and natural language processing tasks [38].

### Inclusion Criteria

Over 200 applicants who were interested in our four-week smoking reduction and cessation interventions were screened based on their self-reported characteristics. The inclusion criteria were regular smokers (smoking 5 days per week) who were between the ages of 18 and 65 years and living in the United States. To be eligible, participants had to have no chronic disease interfering with their daily lives, no use of illicit drugs, and be motivated to quit smoking (> 80, on a 100-point motivation to quit smoking scale [39]). To ensure that access to and use of Facebook were not barriers to participate in the intervention, participants had to have Internet access and had to use Facebook through their mobile or computer devices on a regular basis.

Qualified applicants (N=132) were invited to participate in our study. The eligible participants who responded to our invitation (N=46) were randomly assigned to one of the following conditions: email condition, MTurk-only condition, or the Facebook condition. Participants were introduced to their intervention and coached on how to participate in it. This report focuses on the subjects who were randomized to the Facebook condition (n=16) in order to give special attention to the findings that are unique due to the social media features exclusively available on Facebook (eg, comments, share, likes, and wall postings). Primary outcomes from all three conditions will be published in a separate report.

### Intervention Guidelines

Participants were first provided with an electronic informed consent form. Before the start date of the intervention, researchers contacted consenting individuals through an individual phone call meeting and provided guidelines in greater detail on how to participate in the Facebook intervention. For example, participants were encouraged to share their thoughts, progress, and peer support. We also provided practical methods on engaging with the intervention materials on a daily basis by leaving comments, liking posts, and interacting with other peers in the group throughout the four-week intervention period. The participants were informed that there is no incentive for intervention engagement in our smoking reduction and cessation Facebook group. We informed participants that our research members would post different antismoking messages throughout the intervention period and provide social support to keep participants motivated to engage in action for smoking reduction and cessation. In addition, on the start date, the research team greeted all participants on the Facebook group wall by posting encouraging statements such as “…If you are having a hard time quitting, let us hear. We are here to support you and encourage you to achieve your goal.” This greeting statement was used to set a positive tone and an atmosphere inclusive of all participants. All procedures, materials, and study protocols were reviewed and approved by the university’s Institutional Review Board.

### Research Design of the Smoking Reduction and Cessation Facebook Group

We implemented and targeted different levels of message exposure and engagement and social support systems over four weeks. During week 1 (high message exposure combined with low engagement and social support), we posted antismoking messages three times per day without directly encouraging people to respond to the materials or share their thoughts. During week 2 (low message exposure combined with high engagement and social support), we posted antismoking materials once per day and delivered supportive comments and fostered user engagement by directly asking participants to share their motivating factors, their thoughts on posted antismoking messages, and their progress on quitting smoking with the group. During week 3 (high message exposure combined with high engagement and social support), we posted antismoking messages three times per day. In addition, a professional clinical expert joined the Facebook group and provided guidance on smoking reduction and cessation as well as methods to cope with nicotine withdrawal. We also continued our targeted engagement and social support communications by asking people to share their thoughts toward the guidance. During the last week (low message exposure and low engagement and social support), we posted antismoking materials once per day that focused on mindfulness, self-regulatory tips, and resources for smoking reduction and cessation. Participants were blinded from the intention of the intervention designs regarding message exposure frequency and levels of engagement and social support systems.

### Stimulus Materials for Antismoking Message Exposure

In order to prepare intervention materials to be disseminated on our smoking reduction and cessation Facebook group for four weeks, a total of 80 different antismoking advertisements, campaign messages, and news articles were collected from publicly available online sources, such as smokefree.gov, cancer.gov, and the CDC’s Media Campaign Resource Center (MCRC), a rich database with more than 10,000 antismoking ads produced by different states and federal agencies. The collected antismoking materials were either video-based or text-and-image-based materials that have shown population-level success or promising evidence on promoting tobacco control and prevention (eg, the “Tips From Former Smokers” campaign). To select the final set of intervention materials, in a separate MTurk-based randomized experiment, we evaluated the relative effectiveness of 80 antismoking materials among 1288 smokers prior to the interventions. Based on composite scores of message effectiveness and post-antismoking attitudes toward randomly assigned antismoking material, a total of 56 antismoking messages out of the 80 units were selected as intervention materials (3 messages × 7 days for the first week, 1 message × 7 days for the second week, 3 messages × 7 days for the third week, and 1 message × 7 days for the last week).

The 56 units of antismoking messages were posted in a random order on our smoking reduction and cessation Facebook group. Based on the ongoing feedback from our participants and weekly surveys, 5 message units of these 56 (approximately 9%) were replaced with other antismoking materials to correspond to the needs of participants (eg, asking for more information on smoking cessation tips).

**Systematic Delivery of Evidence-Based Antismoking Materials**

Our smoking reduction and cessation Facebook group intervention started in late November 2015 and ended in early January 2016. We delivered antismoking materials with different frequencies across four intervention weeks (as described above) but with fixed time schedules: 8:00 AM, 12:00 PM, and 5:00 PM (Pacific Time) for the first and third week; and 11:00 AM (Pacific Time) for the second and fourth week.

**Engagement and Social Support Systems via Social Media Features**

In addition to using social media as an intervention modality where participants were exposed to antismoking messages frequently, the research team utilized communication features on the Facebook group, such as pressing the “like” button to express support and affective responses toward users’ wall postings and comments and leaving “comments” to provide constructive feedback. These activities were implemented to synchronously reciprocate them with information and foster social support and user engagement.

### Research Assessments

A baseline survey, all weekly surveys administered during the four-week intervention period, and a two-week follow-up survey were conducted to participants via MTurk by using the “qualification type” function on MTurk. This function made the survey available only to our intervention participants. Participants were compensated with US $8 for each baseline and weekly survey and US $15 for the two-week follow-up survey for a total of US $55 over the study period. The median values of the time spent by participants on survey assessments throughout the entire intervention period ranged between 4.21 minutes and 13.96 minutes.

### Demographic and Smoking Characteristics at Baseline

Demographic information such as age, gender, marital status, ethnicity, and race, self-reported smoking status (the average number of cigarettes participants smoked in the past 7 days), motivation to quit [39], and behavioral intention to quit smoking [40] were assessed at baseline. The readiness to quit smoking was also measured on a 10-point Likert scale [41], where scores between 1 and 3 reflect low readiness to quit (eg, “I don’t want to quit. Tobacco is not a problem for me.”); the range between 4 and 7 indicates moderate readiness to quit (eg, “I know quitting would be good for my health. I am interested in advice about quitting.”); and the scores between 7 and 10 indicate high readiness to quit (eg, “I am ready to quit using tobacco. I would like help to quit using tobacco.”).

### Data Analysis: Facebook ESSC Scores, Smoking Reduction and Cessation

#### Primary Outcomes

The primary outcome was self-reported smoking reduction reported at baseline and the last follow-up (adopted from [42,43] and modified for the study). The number of cigarettes participants smoked per week measured at follow-up was subtracted from that measured at baseline to compute the reduced number of weekly cigarettes consumed per participant. Another primary outcome was smoking cessation (7-day point smoking abstinence at the follow-up).

#### Predictor Variable (Facebook ESSC [/ˈesit/] Scores)

For the key independent variable, we constructed individual-level Facebook engagement and social support composite scores (referred to as “Facebook ESSC Scores” hereafter) to capture user engagement and the social support received from our Facebook group. The Facebook ESSC score was aggregated for each participant by combining the number of postings each participant generated (both wall postings and comments) and the number of “likes” each participant received during the intervention period. Two trained coders verified the number of likes each participant received and the number of comments or wall posts each participant made. The two coders reached a consensus on these results.

#### Secondary Outcomes

Secondary constructs measured at baseline, weekly, and follow-up surveys include the antismoking attitudes scale on a 7-point semantic differential scale [44], readiness to quit on a 10-point Likert scale [41,45], motivation to quit [39], self-efficacy beliefs on a 7-point Likert scale (adopted from Wei et al study [46] and modified for the study), and perceived social support on a 5-point Likert scale [47]. In all of these scales, higher values indicate positive attitudes toward smoking, greater readiness to quit smoking, greater belief in self-efficacy, and greater perceived social support, respectively.

#### Facebook Intervention Feasibility Inventory

We generated the Facebook Intervention Feasibility Inventory by adopting and modifying questionnaires from usability and acceptability scales that were validated in the mHealth intervention context [48-50]. The Facebook Intervention Feasibility Inventory was designed to measure the perceived feasibility of using a Facebook group for smoking reduction and cessation interventions with 24 randomly ordered items on a 7-point Likert scale at follow-up. Examples of responses include statements such as “I thought the anti-smoking Facebook Group was easy to use.” and “I felt very confident using the Anti-smoking Facebook Group.”

#### User Engagement Patterns and Message Exposure Tracking

We monitored participants’ engagement with the intervention content and other members by looking at the frequency of postings and “likes” that participants generated on the Facebook group. Due to limited access to extract user data specific to “seen by” activities, our trained research members counted the number of “seen by” activities per post. We also unobtrusively observed participants’ exposure to intervention messages by checking the “seen by” feature on a daily basis, which enabled the research team to track whether each user had seen the materials posted on the wall of our Facebook group.

#### Statistical Analysis

R package version 3.2.5 [51] and IBM SPSS statistics version 22 [52] were used for statistical analyses. Descriptive analyses were first performed on the demographic variables, predictors, and primary and secondary outcomes to summarize distributions and patterns of each construct. For the primary outcome, we ran adjusted regression analyses to examine the relationship between the predictor and the primary outcome variable while controlling for baseline covariates (gender, readiness to quit, and smoking status, ie, the number of cigarettes participants smoked in the past 7 days at baseline). For secondary outcome variables, we performed General Linear Model (GLM) analysis with repeated measures to explore the main effects of Facebook ESSC scores on repeated secondary outcomes while controlling for the same baseline covariates. We performed a confirmatory factor analysis with an oblimin rotation on the Facebook intervention feasibility questionnaires. A bivariate correlation matrix was computed to examine reliability coefficients between predictors, smoking reduction, and Facebook feasibility subfactors. We also used various R packages and publicly available online software for data visualization to demonstrate participants’ engagement trends based on the total number of postings aggregated throughout the intervention period.

## Results

### Demographic and Smoking Characteristics at Baseline

The majority of participants were white (n=15, 94%), female (n=13, 81%), and between 25 and 50 years old (mean 34.75, SD 8.15). On average, participants smoked 11.31 cigarettes per day (SD 6.81) and 6.93 days per week (SD 0.25) at baseline. The degree of readiness scores for smoking cessation was 7.50 (SD 1.59), which indicates high readiness to quit smoking. During the past 12 months prior to baseline, participants stopped smoking 1.81 times (SD 1.47 times) for at least one day or longer. The 7-item antismoking attitude scale at baseline had a high inter-reliability (Cronbach alpha=.91) and loaded on a single confirmatory factor (with an eigenvalue=4.965 with 70% variances being explained by this one factor). Thus, we created a composite score (mean 2.37, SD 1.22): lower values indicate strong antismoking attitudes, such as smoking cigarettes is “bad, useless, and harmful for my health”; and higher values indicate positive attitudes toward smoking, such as smoking cigarettes is “good, beneficial, and useful.”

### Facebook ESSC Scores, Smoking Reduction and Cessation

All participants (N=16) completed all 6-time-point surveys. Descriptive statistics of predictors (Facebook ESSC scores), primary and secondary outcomes, and Facebook intervention feasibility questionnaires are presented in Table 1. Compared with the baseline, participants reported smoking an average of 60.56 fewer cigarettes per week (SD 38.83) at the follow-up, and 25% of the participants reported 7-day point smoking abstinence at the follow-up. Facebook engagement and social support composite scores (Facebook ESSC scores) were generated (mean 19.19, SD 24.35). The final adjusted linear regression model revealed that a one-unit increase in Facebook ESSC scores predicted a 0.56 unit decrease in cigarettes consumed in the past 7 days (standard error, SE=.24, *P*=.04, 95% CI 0.024-1.09) when baseline covariate characteristics were controlled, *F*_4, 11_=8.85, *P*=.002, adjusted R^2^=.68.

### Composite Scores of Secondary Outcomes and Exploratory Analyses

Seven-item antismoking attitudes showed a high reliability for each time-point survey, ranging from Cronbach alpha=.91 to .99. Thus, we created a composite score for each time-point. Similarly, 5-item self-efficacy questionnaires had a high reliability for each time-point survey, ranging from Cronbach alpha=.88 to .98. We generated a composite score for self-efficacy beliefs measured at each time-point of the surveys. For perceived social support questionnaires, five items were averaged to a single factor for each time-point survey after verifying a good reliability score for each survey (Cronbach alpha scores ranged from .84 to .92). The descriptive statistics of secondary outcomes are reported in Table 1. Exploratory GLM analysis with repeated measures revealed that Facebook ESSC scores were not significant predictors of secondary outcomes when the baseline covariates were adjusted (*Fs*<1).

**Table 1:** Predictors, primary and secondary outcomes, and Facebook feasibility.

Variables		Statistics					
**Predictors**		Mean (SD)					
	Number of Facebook “likes” received	13.25 (17.67)					
	Number of Facebook “comments/wall posts” generated	5.94 (6.96)					
	Number of Facebook engagement scores	19.19 (24.35)					
		Baseline	Week 1	Week 2	Week 3	Week 4	Follow-up
**Primary outcomes**							
	Mean number of cigarettes smoked per week among smokers mean (SD)	79.19 (47.66)	44.38 (60.09)	32.44 (44.42)	29.81 (41.84)	20.44 (36.51)	18.63 (35.33)
	Number of people who quit smoking in the past 7 days, n (%)	0 (0)	0 (0)	2 (13)	3 (19)	3 (19)	4 (25)
**Secondary outcomes, mean (SD)**							
	Mean Antismoking attitude scale score	2.37 (1.22)	1.76 (1.33)	1.73 (1.34)	1.66 (1.32)	1.60 (1.51)	1.60 (1.53)
	Mean self-efficacy for smoking cessation scale score	–	6.06 (0.70)	5.83 (0.83)	6.18 (0.75)	6.40 (0.67)	6.05 (1.50)
	Mean score on readiness to quit item	7.50 (1.59)	8.19 (1.22)	7.88 (1.59)	7.94 (1.53)	8.63 (1.20)	8.56 (1.41)
	Mean perceived social support scale score	–	3.95 (0.87)	4.10 (0.64)	4.03 (0.79)	4.18 (0.84)	3.89 (0.88)
**Facebook intervention feasibility questionnaires, mean (SD)**							
	Response efficacy (alpha =.96)	–	–	–	–	–	5.47 (1.20)
	Perceived technology barriers (alpha=.97)	–	–	–	–	–	2.23 (1.45)
	Easiness to use (alpha=.96)	–	–	–	–	–	6.02 (0.98)

**Table 2:** Correlations across predictors, smoking reduction, and Facebook feasibility subfactors. Facebook engagement and social support composite scores (Facebook ESSC scores) are combined values of the number of Facebook “likes” one received (1 in the table) and the number of Facebook “comments” and “wall postings” each person generated (2 in the table).

Variables		1		2		3		4		5		6	
1	Number of Facebook “likes” received		–										
2	Number of Facebook “comments/wall posts” generated		.95^a^		–								
3	Facebook ESSC scores		.996^a^		.97^a^		–						
4	Reduced number of cigarettes smoked		.49^c^		.48		.49^c^		–				
5	Facebook response efficacy		−.19		.01		−.01		.14		–		
6	Perceived technology barriers		−.34		−.35		−.34		−.56^b^		.12		–
7	Easiness to use		.24		.20		.23		.35		.62^b^		−.15

^a^*P*<.01.

^b^*P*<.05.

^c^*P*=.05.

### Factor Analysis on Facebook Intervention Feasibility Questionnaires

A confirmatory factor analysis with a direct oblimin rotation was performed on the 24-item Facebook intervention feasibility questionnaires. Items within each subfactor with loading scores greater than 0.6 were averaged to compose three subconcepts under the umbrella concept of perceived Facebook feasibility for health interventions. Those subfactors represent Facebook response efficacy (alpha=.96), perceived barriers of using Facebook (alpha=.97), and easiness of using Facebook for smoking reduction and cessation interventions (alpha=.96), respectively (Table 1 for descriptive analyses). Bivariate correlation coefficients between these feasibility subfactors and predictors and primary outcomes are presented in Table 2. Participants with low perceived barriers of using the Facebook Group tend to have greater reduction in weekly cigarette smoking compared to those with high perceived barriers of using the Facebook group intervention (Pearson *r*=−.56, *P*=.02).

### User Engagement Patterns and Message Exposure Tracking

**Figure 1:**
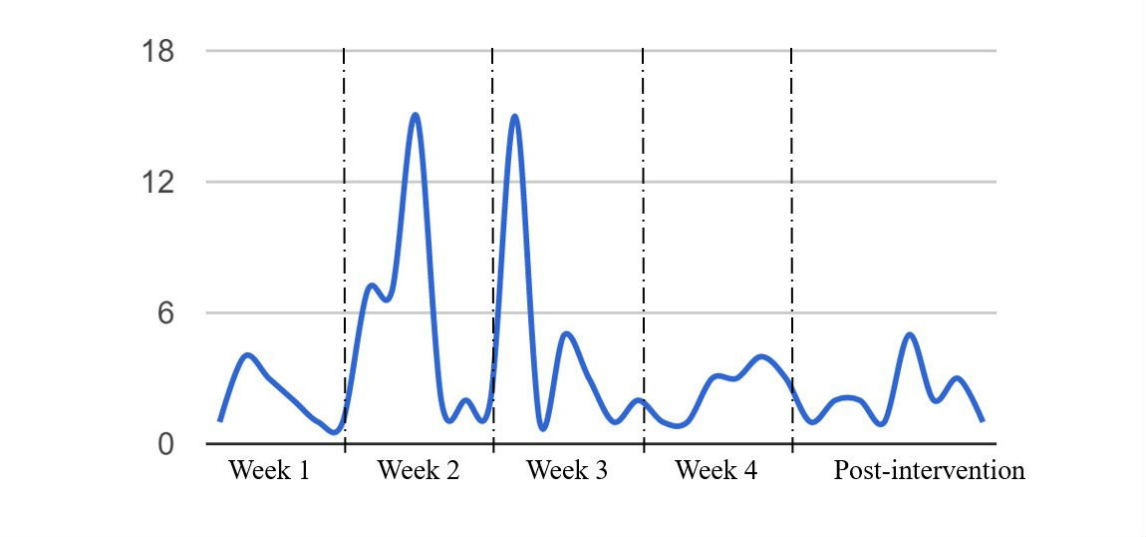
User engagement during the four-week intervention period. The y-axis indicates the number of wall posts and comments participants generated within the smoking reduction and cessation Facebook group. The values are indicative of user engagement.

Figure 1 presents the aggregated number of postings from participants across the four-week intervention period. The second and third weeks, in which a high level of engagement and social support systems were targeted, demonstrate an increased generation of comments and wall posts by the participants during these two weeks. We proposed that the number of comments and wall posts indicated active user engagement. Note that week 1 and week 4 (low engagement and social support systems) revealed a decreased number of comments and wall posts in the graphic pattern, indicating relatively passive user engagement. After the intervention was over, we received a few posts from participants reporting their success in maintaining smoking cessation, as well as thanking the researchers for their help and requesting further assistance about tips for completely quitting smoking.

## Discussion

### Principal Findings and Comparison With Prior Work

Using a closed Facebook group, we developed and delivered a smoking reduction and cessation intervention in a cost-effective manner while overcoming geolocational barriers and time constraints. We found that Facebook was highly feasible and we demonstrated 100% study retention and survey completion rates. In this study, 25% of participants reported 7-day smoking abstinence at the follow-up, and those who continued had dramatically reduced the number of cigarettes they smoked weekly. These outcomes are consistent with and comparable with previous studies of Facebook-delivered interventions for smoking [53].

Although we promoted smoking cessation as an optimal outcome for our intervention, we also accepted smoking reduction as a positive form of behavior change. Helping smokers to cut down on cigarette use has been attempted in many controlled trials [54]. However, we acknowledge that smoking reduction should be considered as an intermediate precursor to quitting rather than a goal of the intervention because reduced- and light-smoking still carry a considerable level of health risk for cardiovascular disease and cancer, as observed among heavy daily smokers [55]. That said, smoking reduction can have a substantial health impact in the long run [56,57]. For example, in a 30-year longitudinal follow-up cohort study, Godtfredsen and colleagues [58] found that a 50% smoking reduction significantly decreased lung cancer risk among heavy regular smokers.

Results also suggest that engagement and receipt of social support within this Facebook health communications intervention predicted smoking reduction among motivated smokers. Specifically, a one-unit increase in Facebook ESSC scores predicted a 0.56 unit decrease in cigarettes consumed in the past 7 days. That is, participants who received more “likes” and those who posted more content on our Facebook group, indicative of social support and user engagement, were more likely to reduce their weekly smoking (*F*_4,11_=8.85, *P*=.002, ΔR^2^=.68), suggesting a potential mechanism of action for the intervention.

We generated the term “Facebook engagement and social support composite scores” (ESSC [/ˈesit/] scores) in this study and tested the predictive validity of the ESSC scores on smoking reduction. The composite score was based on our conceptualization that “writing comments and wall posts” is an indicator of user engagement, as also defined by Facebook [59]. We used the “like” feature on Facebook to express our positive reaction toward participants and form perceived social support. The number of likes participants received from the interventionists and other participants within the intervention Facebook group was conceptualized as an index for receiving social support. However, we acknowledge that receiving comments (reciprocity) is not only an indicator of user engagement, but also an indicator of social support (as perceived social support increases when self-disclosed information is reciprocated [60]). That is, these two constructs (user engagement and social support) are covarying and correlated constructs as we found in our study (Pearson *r*=.95).

Given this conceptualization, we were interested in how the user engagement and social support systems worked synergistically to enhance intervention outcomes. Determining whether user engagement is exclusively more important than perceived social support or vice versa for predicting smoking reduction was beyond the scope of our research. Thus, we proposed a composite score by combining them to serve our conceptual approach. In addition, our approach was aligned with the principle from test theory that composite scores are more reliable than individual items [61]. Note that the number of likes participants received from peers and interventionists (beta coefficient=.72, SE=.33, *P*=.05) as well as the number of comments and wall posts each participant generated (beta coefficient=2.05, SE=.78, *P*=.02) were independently significant predictors of the reduced number of cigarettes consumed in the past 7 days. We suggest that future work disentangle the unique value of these two constructs and build a prediction model focusing on each construct as a single predictor. We also encourage future research to validate and examine the replicability of this composite score.

Throughout the intervention period, on average, participants generated six comments during the four-week intervention period (SD 6.96, median 3.50). Thrul and colleague [62] found that 79 participants made a total of 718 individual comments during the three-month intervention period, which are about nine comments per participant for three months. When averaged by month, their engagement level is three comments per participant per month. We consider our user engagement level (six comments or wall posts per participant for four-week) is comparable with other social media–based interventions for smoking. In fact, we found that the average number of comments and wall posts were negatively skewed due to observers who did not generate any comments or wall posts (n=6, 37.5%). This proportion of observers is relatively low compared with that reported in Thrul et al’s study [62]. On social media health forums, lurking or observing is a common practice [63,64]. In future studies, we hope to develop and examine engagement strategies specific to targeting these intervention observers.

To understand how and for whom social media–based interventions work, future work may examine potential moderating factors that impact the relationship between user engagement and intervention outcomes. An array of baseline characteristics have predicted technology-based intervention outcomes [65]. These characteristics may include demographic characteristics, personalities and traits (eg, self-regulation [66]), and even the stage of change for smoking cessation [62]. By investigating moderating factors in future research, researchers may identify subgroups of smokers that may benefit the most from social media–based interventions for smoking reduction and cessation.

We operationalized two key intervention components to maximize the persuasive effects of social media platforms in promoting smoking reduction and cessation: (1) exposure to antismoking messages and (2) participant engagement and social support systems. Prior studies have demonstrated that exposure to health campaign messages can enhance health behavior by changing one’s beliefs about expected health outcomes [67] and by providing educational messages to increase necessary knowledge and skills [22]. Message exposure was successful as most of the posted materials were “seen by” almost everyone throughout the intervention weeks, although there was a slight decrease in message exposure during the last week. The diminished message exposure was not surprising but rather consistent with previous social media–based health interventions [62,68].

CDC’s Best Practices Report, released in 2014, recommends the reuse of existing advertisements and campaign messages rather than producing new content in order to reduce the cost, time, and untested risks associated with developing new ones [1]. In this regard, we focused on systematically delivering evidence-based antismoking messages from existing campaigns and advertisements, which not only helped us save on time and cost but also enabled us to readily examine how message exposure and engagement with the intervention content led to smoking reduction.

At the time we developed this study, there was no standardized, evidence-based model or framework applicable to designing Facebook group–based interventions for smoking reduction and cessation. Thus, based on prior health communication and technology literature, we developed an intervention model using two main components: “persuasive message exposure” and “supportive engagement systems.” We used a varied frequency of message exposure (three times per day or one time per day), as there was no empirical evidence on the optimal dose of message exposure for a social media–based intervention. We randomly juxtaposed these two components (high vs low message exposure frequency × high vs low engagement and social support) to develop our intervention model. This randomly juxtaposed combination led to four weekly designs, including high message exposure and low engagement and social support systems for week 1; low message exposure and high engagement and social support systems for week 2; high message exposure and high engagement and social support systems for week 3; and low message exposure and low engagement and social support systems for week 4. Our findings should be understood with caution. We did not examine which weeks resulted in the most successful intervention outcome (smoking reduction), but we tested the overall impact of the intervention as a whole (before and after the intervention) on smoking reduction. Thus, the risk of any possible confounding effect due to the varied frequency of message exposure is minimized because we did not test smoking reduction by individual week.

An interesting finding about user engagement is that although we manipulated the frequency of message exposure, there was no resulting effect on increasing user engagement, as shown in Figure 1. The targeted engagement and social support systems directly influenced user engagement, not the message exposure frequency. The distinctive weekly patterns shown in Figure 1 highlight the notion that in order to foster active user engagement, a significant predictor of smoking reduction, interventionists should directly target user interactivities. For example, the second week delivered a high level of user engagement by directly asking personally relevant questions and encouraging participants to share their progress with others in the Facebook group [69] (eg, “What motivates you to quit smoking?” and “Have you reduced the number of cigarettes today?”). Note that week 2 and week 4 had the same messaging frequency: antismoking message was posted once a day during these two weeks. When controlling for the messaging frequency as once per day, the number of user comments and wall posts (indicative of user engagement) in week 2 was higher than that of user comments and wall posts during week 4. This pattern indicates that interventionists should specifically target user engagement in addition to posting antismoking messages. We encourage researchers to adopt and apply effective persuasion tactics and principles to strategically target user engagement within social media–based health interventions.

Another strength of the study was 100% study retention throughout the MTurk-linked surveys at six different time points. Various technology features on MTurk, such as qualification assignment and the online payment system allowed us to conduct longitudinal surveys. We demonstrated that MTurk can be a platform for a wide range of research activities, ranging from recruitment of smokers living in the United States to multiple times of follow-up assessments with participants.

### Limitations and Future Directions

Our findings successfully demonstrated the feasibility of social media technologies to offer smoking reduction and cessation interventions with the strategic delivery of engagement and social support systems. Our findings, however, should be understood within the limitations imposed by research budgets and the scope of the study. We did not objectively verify self-reported abstinence; thus, it is possible that the impact of the intervention may be inflated. The reported outcome on the reduced number of cigarettes per week does not correspond to the same level of reduction in toxicant exposure. In future technology-based interventions for smoking reduction and cessation, researchers should embrace practically feasible methods for measuring objective markers of nicotine toxicology [70].

Our sample size in this study was relatively small. Thus, rather than using a complex modeling approach such as latent growth curve modeling, we simplified our statistical model and directly examined the predictive value of Facebook-mediated engagement and social support in explaining smoking reduction outcomes. The dataset of 16 participants with no missing data still provided enough statistical power to detect the effect of the primary regression model outcomes.

Another limitation is that the gender and race of our sample were relatively homogeneous, mostly white women. In future research, we hope to replicate the interventions with bigger sample sizes and involve participants with characteristics that are more heterogeneous than the current sample to establish the generalizability and reproducibility of the findings. With an increased sample size, future studies should examine pathways of intervention processes with intermediate factors, such as enhanced self-efficacy and perceived social support, to reflect the dynamics of behavior change [71,72]. Also, Figure 1 shows different descriptive patterns by week. We did not examine a statistical difference using repeated measures by week on the number of wall posts and comments (user engagement). In future research, we encourage to examine statistical difference on this engagement matrix, and perhaps to design multiple Facebook groups to prevent any potential confounding or spillover effects across weeks.

We examined the feasibility of the communication features in our Facebook group that were utilized to deliver theory-guided intervention components such as message exposure and engagement and social support systems among the optimal set of participants (self-motivated participants who wanted to quit smoking at baseline). In future work, another important question might be whether social media–based interventions can have a significant impact on enhancing these intermediate factors (eg, enhancing motivation to quit, and pro-quitting attitudes), even among those with low motivation to quit smoking at baseline. If social media–based interventions can successfully enhance those factors and smoking reduction and cessation among participants with low motivation, the expected significance of the interventions can be much greater than this study.

After our four-week intervention, followed by a two-week follow-up survey, we learned that participants continued to use our Facebook group and some participants expressed that they wanted the interventions for a longer period of time. Social media platforms provide novel opportunities to operationalize persuasive technologies for scalable interventions and to maintain active engagement and long-lasting intervention outcomes [18]. Future efforts in this line of research may examine and identify which communication and intervention strategies are most effective in sustaining active user engagement and maintaining long-lasting social ties for supportive networks. Additionally, there are several challenges and questions to consider for the future implementation of this work, including finding an optimal dose and information balance between support providers and support recipients and protecting the privacy of online intervention participants, especially for a large-scale intervention.

As reviewed, theory-driven and evidence-based interventions using Facebook for health promotions are promising. A growing line of research has shown positive effects of Facebook use on various health outcomes, from smoking cessation [62,73] and physical activities [74] to sexual health promotion [9,75]. The benefits of social media, however, go beyond its technological affordability, scalability, and accessibility. In fact, social media use provides psychological benefits that are essential to fundamental human needs. Researchers from various fields have examined psychological benefits and gratification from using Facebook [60,76-81], such as enhanced self-esteem and psychological well-being [78], increased social capital [79], and refinement of self-affirming values [77]. Furthermore, technology features of Facebook facilitate self-disclosure and reciprocal interactions with others, and these activities have been found to be intrinsically therapeutic and rewarding for humans [82]. Although targeting these psychological benefits were not within the scope of our study, and thus were not measured nor manipulated, we hope future work will consider how to actively facilitate these psychological benefits when using Facebook as an intervention tool for health promotions.

### Conclusions

This study is the first Facebook-mediated intervention research that systemically promoted antismoking communication strategies and social support and engagement systems as mechanisms of behavior change within a Facebook group. We conceptualized Facebook “likes” and “wall postings and comments” as the manifestation of social support and user engagement. Our findings imply that receiving one Facebook “like” or posting on the Facebook group at least once predicts almost one less cigarette in the past 7 days. The study supports positive effects of Facebook-mediated communication, engagement and social support systems for smoking reduction and cessation, and highlights the public health potential of social media interventions for scaling-up tobacco control and prevention efforts. It also provides practical guidelines for designing communication strategies and persuasive, social media–based smoking reduction and cessation interventions that might be useful for future research.

## Abbreviations

Facebook ESSC Score: Facebook engagement and social support composite score
